# Trace Elements as Contaminants and Nutrients

**DOI:** 10.3390/foods11091337

**Published:** 2022-05-04

**Authors:** Cristina Couto, Edgar Pinto, Agostinho Almeida

**Affiliations:** 1 TOXRUN–Toxicology Research Unit, University Institute of Health Sciences, CESPU, CRL, 4585-116 Gandra, Portugal; 2 LAQV/REQUIMTE, Department of Chemical Sciences, Faculty of Pharmacy, University of Porto, Rua de Jorge Viterbo Ferreira 228, 4050-313 Porto, Portugal; ecp@ess.ipp.pt (E.P.); aalmeida@ff.up.pt (A.A.); 3 Department of Environmental Health, School of Health, P. Porto, 4200-072 Porto, Portugal

Knowledge about trace elements has evolved remarkably in recent decades, both in terms of their metabolism and their functions. Acting mainly as cofactors of enzymatic systems, several trace elements play an essential role in numerous physiological processes in the human organism, from cell metabolism to the immune response and gene expression, among others. On the other hand, it is also well known that excessive exposure to trace elements can be highly harmful and even fatal.

The benefits of trace elements but also their impact as potential toxicants in the food sector was the topic chosen for this Special Issue of the *Foods* journal, entitled “Trace Elements as Contaminants and Nutrients”. It comprises seven research papers [1,2,3,4,5,6,7] and one review article [8], addressing precisely those two main topics: trace elements as contaminants and trace elements as nutrients.

Konieczynski et al. [1] investigated the potential role of trace elements in the claimed antidiabetic effect of some medicinal plants. For this purpose, they compared the trace element content of infusions prepared from medicinal plants traditionally used in the treatment of diabetes and a control group (medicinal plants without this therapeutic indication). The results do not support an important role of trace elements in the antidiabetic effect of the studied plants.

The nutritional composition of *nfuma*, a traditional flour obtained from the fruits of *Strychnos madagascariensis* (a tree from tropical and sub-tropical Africa), as prepared by populations in Mozambique, was evaluated by Chemane et al. [2]. The authors determined the content of sugar, amino acids, vitamin E, carotenoids, macrominerals (Ca, Mg, Na, K) and a wide panel of trace elements (Fe, Li, Be, Al, V, Cr, Mn, Co, Ni, Cu, Zn, As, Se, Rb, Sr, Cd, Cs, Ba, Tl, Pb, Bi) in *nfuma* from four districts of Mozambique, and evaluated its nutritional adequacy, according to current nutritional recommendations. Safety issues, related to possible exposure to potentially toxic trace elements, were also addressed.

The study conducted by Bielecka et al. [3] aimed to determine the content of selected macrominerals and essential trace elements (Ca, Mg, Cu, Fe, Mn, Se and Zn) in rice and rice products in order to as assess their importance as source of these nutrients in the adult European population diet. All products studied proved to be a source of Cu, Mn and Se, while most could also be considered a source of Mg and Zn. Not surprisingly, significant differences in the levels of the studied elements were observed between processed and unprocessed products.

The results from six years (2015–2020) of official control of the Cd, Pd and Hg content in imported whole durum wheat (from Australia, Canada, Kazakhstan, Russia, Turkey and the USA) into the Italian market are reported by Pompa et al. [4]. Data are discussed according to seasonality, year and country of origin. The trend observed in other studies at a global level of a decrease in Pb, Cd and Hg levels in recent years was confirmed. However, durum wheat products continue to represent important sources of exposure to Cd and Pb. 

The effect of different food processing (high-pressure, freezing, frozen storage, canning) on the content of macrominerals and a wide panel of trace elements (Na, K, Mg, Ca, Ba, Mn, Fe, Co, Cu, Cd, Sn, Pb, P, As, S, Se) in brine-canned mackerel (*Scomber colias*) was studied by Prego et al. [5]. Content changes are tentatively explained. 

In another work, by Garcia-Galdeano et al. [6], the content of Zn, Cu and Fe in several dehydrated aromatic plants (thyme, rosemary, cloves, oregano and basil) was studied, as well as their microbiological quality (assessed by plate counts of L. monocytogenes and other foodborne pathogens). The growth of several of the foodborne pathogens tested was positively correlated with the levels of Zn, Cu and Fe, which the authors attributed to the fact that those trace elements could act as a "growth factor".

Dahl et al. [7] studied the effect of different food processing (boiling, pan-frying, oven-baking) on the iodine and mercury content of Atlantic cod (*Cadus morhua*). Boiling has been shown to significantly decrease the iodine content (approx. 10–20%), so the authors suggest that this be specified in food composition databases as it may influence the estimation of the actual iodine intake [7].

Lastly, a mini review summarizes recent developments on the use of metallic nanoparticles in the food science and technology sector, including toxicity/biosafety and regulatory issues [8].

Overall, the papers published in this Special Issue highlight the importance and need for further research on trace elements in the food sector. As guest editors, we sincerely hope that readers will find it interesting and informative, and we thank all authors for their highly qualified contributions.
